# The effects of commercially available footwear on foot pain and disability in people with gout: a pilot study

**DOI:** 10.1186/1471-2474-14-278

**Published:** 2013-09-24

**Authors:** Keith Rome, Sarah Stewart, Alain C Vandal, Peter Gow, Peter McNair, Nicola Dalbeth

**Affiliations:** 1 Division of Rehabilitation & Occupation Studies, Health & Rehabilitation Research Institute, AUT University, Akoranga Drive, Private Bag 92006, Auckland 1020, New Zealand; 2 Counties Manukau District Health Board, Auckland, New Zealand; 3 Faculty of Health and Environmental Sciences, AUT University, Auckland 1020, New Zealand; 4 Auckland District Health Board, Auckland, New Zealand; 5 University of Auckland, Auckland, New Zealand

**Keywords:** Gout, Pain, Foot, Disability, Joint, Footwear, Comfort

## Abstract

**Background:**

There is limited evidence on non-pharmacological interventions for gout. The aim of the study was to determine whether a footwear intervention can reduce foot pain and musculoskeletal disability in people with gout.

**Methods:**

Thirty-six people with gout participated in a prospective intervention study over 8 weeks. Participants selected one of 4 pairs of shoes and thereafter wore the shoes for 8 weeks. The primary outcome was foot pain using a 100 mm visual analogue scale. Secondary outcomes related to function and disability were also analysed.

**Results:**

The Cardio Zip shoe was selected by 58% of participants. Compared with baseline, overall scores for all shoes at 8-weeks demonstrated a decrease in foot pain (p = 0.03), general pain (p = 0.012), Health Assessment Questionnaire (HAQ)-II (p = 0.016) and Leeds Foot Impact Scale (LFIS) impairment subscale (p = 0.03). No significant differences were observed in other patient reported outcomes including patient global assessment, LFIS activity subscale, and Lower Limb Task Questionnaire subscales (all p > 0.10). We observed significant improvements between baseline measurements using the participants’ own shoes and the Cardio Zip for foot pain (p = 0.002), general pain (p = 0.001), HAQ-II (p = 0.002) and LFIS impairment subscale (p = 0.004) after 8 weeks. The other three shoes did not improve pain or disability.

**Conclusions:**

Footwear with good cushioning, and motion control may reduce foot pain and disability in people with gout.

## Background

Gout is an inflammatory arthritis that occurs due to the deposition of monosodium urate crystals in joints and periarticular tissues [1]. Gout displays a striking predilection to affect the feet, particularly the first metatarsophalangeal joint (1MTPJ), Achilles tendon, midfoot and ankle [2-6]. People with gout experience greater foot pain, impairment and disability than age-matched controls [7]. This degree of foot-related pain, impairment and disability is similar to that reported in people with early and established rheumatoid arthritis (RA) [8,9].

The non-pharmacological management goals for people with foot-related rheumatic diseases are pain management, preservation of foot function and patient mobility [10,11]. One of the therapeutic components that may achieve these goals is footwear. Our group has reported that poorly fitting shoes are linked to foot pain in gout and those with poor footwear have higher foot-related impairment [12]. We further reported that people with gout frequently wear shoes that are either too long or too short, have little cushioning and are more than 12 months old [12]. To date, no research has examined footwear as an intervention for people with gout. The aim of this study was to determine whether a footwear intervention can reduce foot pain and musculoskeletal disability in people with gout.

## Methods

**Figure 1:**
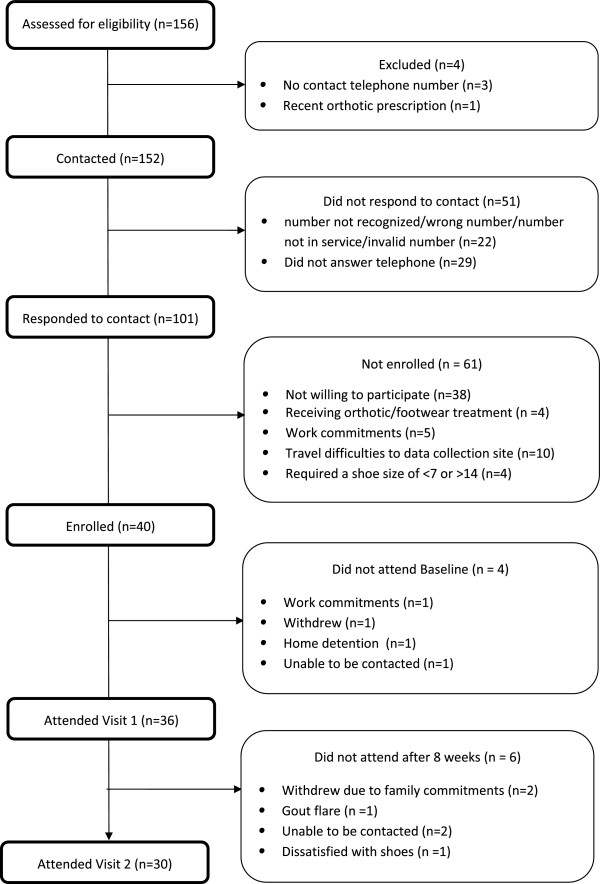
Flow-chart of recruitment of participants.

A prospective intervention study design was utilised. Thirty-six participants were recruited from the rheumatology and podiatric rheumatology clinics based in Auckland District Health Board and Counties Manukau District Health Boards, Auckland, New Zealand (Figure 1). Participants included in the study (i) were over 18 years of age, (ii) had a history of gout according to ACR classification criteria [13], and (iii) were able to walk a minimum of 10 m without the use of a walking aid. People were excluded in the study if they had (i) received any medication for foot pain in the previous 4 weeks, (ii) an acute gout flare at the time of assessment, (iii) history of surgery to the foot, or (iv) received treatment with foot orthoses or footwear within the previous 3 months. The Northern Regional X Ethics approved this study and local institutional approval was also obtained. All participants provided written informed consent before inclusion into the study. The study was registered with the Australian New Zealand Clinical Trials Registry (ACTRN12612000735853).

Based upon data from a previous study examining the primary outcome: foot pain, for a reduction of 20 percentage points, with the power set at 80% and a per comparison significance level of 5%, 36 participants were required [14]. McNemar’s test was used for this determination, and is equivalent to the test of a single footwear pair contrast in the planned analysis. This was deemed sufficient for the purpose of sample size determination.

### Footwear characteristics

**Figure 2:**
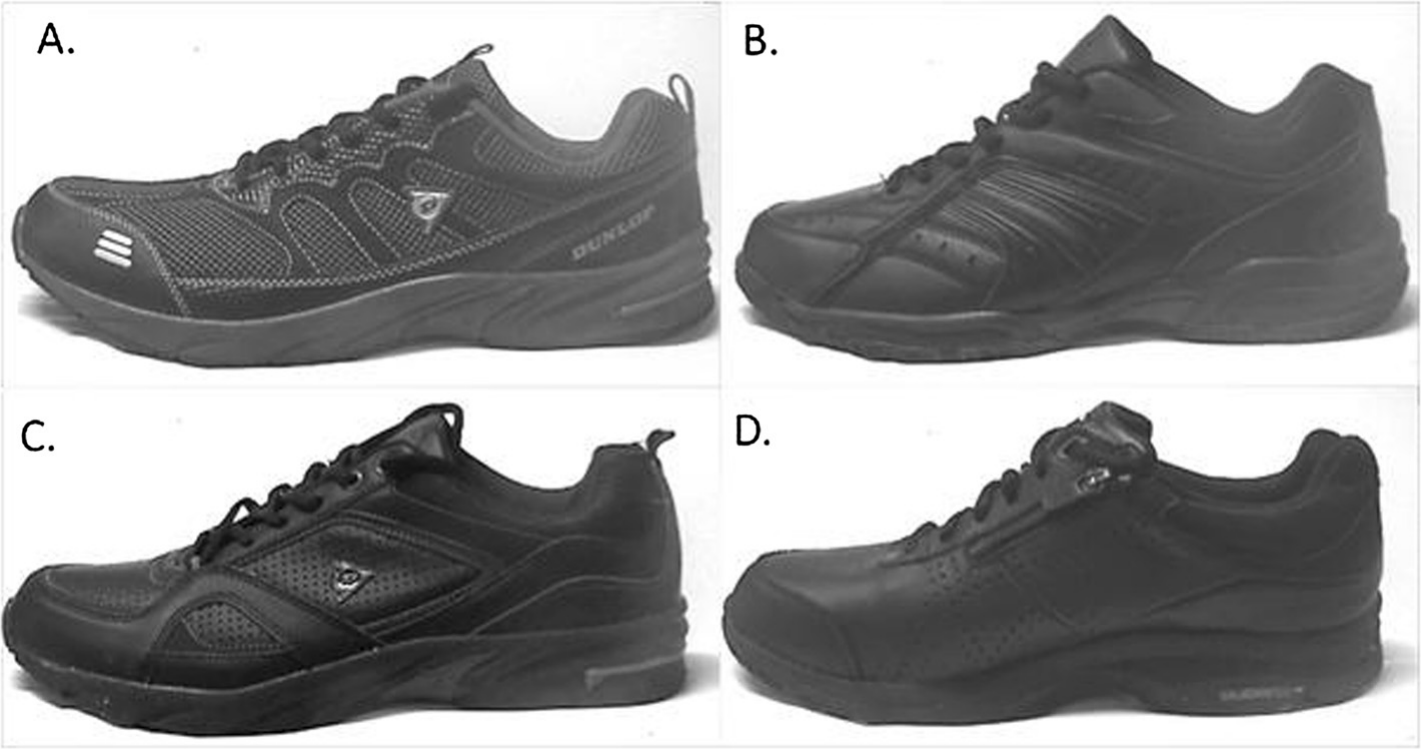
The four study shoes: A. Dunlop Asteroid; B. Helix Viper; C. Dunlop Apollo; D. ASICS Cardio Gel Zip.

Four black, laced walking shoes were selected for the study: Dunlop Asteroid, Dunlop Apollo, Helix Viper and the ASICS Gel Cardio Zip (Figure 2). Footwear characteristics were recorded for each shoe using a previously validated footwear assessment form [15], and are shown in Table 1. These shoes provided variations in footwear characteristics related to shock attenuation, motion control and width. Motion control included fixation of the upper to the foot, heel counter stiffness, and midfoot rigidity [15]. To develop a continuous scale to assess the quality of footwear in relation to motion control properties, each category from the motion control properties items were scored ranging from 0 to 11. Footwear which scores 11 would be considered to possess optimal motion control properties [15].

**Table 1:** **Characteristics of study shoes**^**1**^

**Characteristic**	**Cardio zip**	**Viper**	**Apollo**	**Asteroid**
Upper materials	Leather & Synthetic	Synthetic	Synthetic	Synthetic
Outsole materials	Rubber	Phylon	Synthetic	Rubber
Tread pattern	Textured	Textured	Textured	Textured
Weight (kg)	0.406	0.340	0.353	0.380
Length (cm)	29.5	29.8	29.9	30.0
Heel Height (cm)	3.9	3.9	2.0	2.2
Forefoot height (cm)	2.3	2.6	2.2	2.2
Longitudinal Profile (cm)	1.6	1.3	1.3	1.4
Last (°)	12	11	10	9
Fixation of upper to sole	Slip lasted	Board	Board	Board
Forefoot sole flexion point	At level of MTPJs	Proximal to MTPJ	Level of MTPJs	Distal to MTPJs
Density	Dual	Single	Single	Single
Heel counter stiffness	Rigid	Moderate	Minimal	Minimal
Midfoot sole sagittal stability	Moderate	Minimal	Minimal	Minimal
Midfoot sole frontal stability	Moderate	Minimal	Minimal	Minimal
Motion Control Scale (0–11)	9	4	3	3
Presence of cushioning	Heel/forefoot	Heel/forefoot	None	None
Lateral midsole hardness^2^	Firm (57)	Firm (54)	Firm (56)	Soft (49)
Medial midsole hardness^2^	Firm (53)	Firm (57)	Firm (44)	Firm (53)
Heel sole hardness^2^	firm (56)	Firm (64)	Hard (59)	Firm (65)

^1^All characteristics measured from standard shoe size 11, ^2^Sole hardness measured using a durometer.

At baseline (visit 1), the following clinical features were recorded: the number of foot tophi, the presence and site of subcutaneous tophi, age, gender, ethnicity, body mass index (BMI), disease duration, age of first episode, current pharmacological management, serum urate levels, age at first episode, self-reported flares in preceding 2 months, days off work in last 2 months, employment and history of cardiovascular disease and diabetes. The primary outcome for the study was foot pain using a 100 mm VAS pain scale. Secondary outcome measures included HAQ-II [16], general pain score (100 m VAS) and patient global assessment scale (100 mm VAS). The level of lower limb function was evaluated using the Lower Limb Task Questionnaire [17]. We measured foot disability and impairment using the 2 subscales of the Leeds Foot Impairment Score (LFIS): foot impairment/footwear restriction (LFISIF; range 0–21) and activity limitation/participation restriction (LFISAP; range 0–29) [18].

### Study visits

At visit 1, all participants tried each of the four shoes in a randomly determined order. Randomization involved the presentation of one of a series of sealed envelopes indicating the order in which the footwear was to be assessed by the participant. The exterior of the footwear was masked with tape so the brand name and logos of the shoe would not be recognised. The masking was small so they did not cover any of the design features. In each pair of shoes, participants were asked to complete a circuit that included walking on carpet, on a hard level surface and ascending and descending stairs. To reduce fatigue, each participant was allowed to rest for 5 minutes between footwear. After each circuit, the participant was asked to consider the comfort, fit, style, sole and weight in relation to their normal footwear with a view to choosing a new pair of shoes that they would wear for the following 8 weeks.

At the completion of the baseline assessment, participants were asked to wear the shoes they chose for 8 weeks (visit 2). Each participant was given a diary to complete over this period, and were asked to record the amount of time the footwear was worn each day and to note any adverse events.

### Data analysis

Gender, ethnicity, clinical characteristics such as current pharmacological management, history of hypertension, cardiovascular disease, diabetes, renal impairment were described as n (percentages). All other demographic characteristics were described as mean (SD).

Mixed effects regression was used as the primary analysis of all prospective study outcomes, using participants as random effects, and visit and footwear interaction as fixed effects. Mixed effects (ME) regression is a generalisation of paired t-tests. In particular, the p-values and confidence intervals for footwear-specific changes in Table 2 bear the same interpretation as those that would be obtained from paired-t inference. They are different in value however, as they use all data for full efficiency. ME regression enables the use of Visit 1 data from all participants to test for any specific footwear effect. This stands in contrast to using only the Visit 1 data of participants who selected a particular type of footwear, as would happen with an unmodified paired t-test. In doing so, ME regression still accounts for the association between Visit 1 and Visit 2 measurements for any given type of footwear. ME regression also enables correct inference regarding footwear differences and overall differences regardless of footwear type, all under a single inferential setting.

**Table 2:** Baseline clinical characteristics

	**Value**
Age, years, mean (SD)	57 (13)
Male, *n* (%)	33 (92%)
Ethnicity, *n* (%)	15 (42%) European
	7 (19%) Māori
	9 (25%) Pacific
	5 (14%) Asian
BMI, (kg/m^2^), mean (SD)	34 (8)
Cardiovascular disease, *n* (%)	16 (44%)
Type 2 diabetes, *n* (%)	7 (19%)
Diuretic use, *n* (%)	9 (25%)
Urate-lowering therapy use, *n* (%)	34 (94%)
Colchicine use, *n* (%)	23 (64%)
Prednisone use, *n* (%)	10 (28%)
NSAID use, *n* (%)	22 (61%)
Serum urate, mmol/l, mean (SD)	0.39 (0.13)
Disease duration, years, mean (SD)	15 (11)
Age at first episode, years, mean (SD)	42 (19)
Self-reported flares in preceding 2 months, mean (SD)	3.9 (10.8)
Days off work in last two months, mean (SD)	0.3 (1.5)
In paid employment, n (%)	17 (47%)
Aspirate proven, n (%)	11 (31%)
Foot tophus count, mean (SD)	1.3 (1.8)
Total tophus count, mean (SD)	4.5 (4.1)
Any subcutaneous tophus, n (%)	26 (72%)

The fixed effect structure (visit 1 with no interaction vs. visit 2 in interaction with footwear) was designed to reflect the study design, since baseline measurements were based on participants’ own shoes. Means and their differences were reported as point estimates and confidence intervals. P-values were reported to test the fitted models against three null hypotheses: that of no change between visits for a particular type of footwear; that of equal change between visits for each type of footwear; and that of no change between visits for any type of footwear. Missing outcome data requires no special treatment in mixed regression settings. The significance level for testing was established at 0.05 against two-sided alternatives. Per comparison error rate (no adjustment for multiple testing) was used, but raw p-values presented in all cases. The confidence level for the production of unbiased confidence intervals was set at 0.95. Analyses were performed using SPSS V20.0, R version 2.15.0 for Windows and SAS/STAT software, Version 9.3 of the SAS System for Windows.

## Results

Clinical characteristics at baseline are shown in Table 2. Participants were predominantly middle-aged men, with high rates of obesity and co-morbidities such as hypertension, diabetes and cardiovascular disease. Subcutaneous tophi affecting the 1MTPJ were observed in 12/36 (33%) participants and in the Achilles tendon in 12/36 (33%) participants. Thirty four (94%) participants were on urate lowering therapy, most commonly allopurinol in 29/34 (85%).

High scores for foot pain, disability and impairment were observed at visit 1 (Table 3). Over 50% of the participants’ currently owned shoes were 12 months old or older. Based upon participants’ preference, the Cardio Zip (n = 21, 58%) was the most popular new shoe, followed by the Apollo (n = 7, 19%), then Asteroid (n = 5, 14%) and finally the Viper (n = 3, 8%). Follow-up data including study diaries were available for 30 (83%) participants. Six participants (17%) were lost to follow-up, with 2 participants unable to attend due to family commitments, 1 with gout flare, 1 dissatisfied with shoes, and 2 unable to be contacted. The 30 participants who completed the study diaries wore their chosen shoes for a mean (SD) of 26 (10) hours per week. No adverse events associated with the shoes were reported. During the 8 week trial, one participant commenced allopurinol therapy.

**Table 3:** Differences between visit 1 and visit 2 for patient-reported outcomes

**Parameter**	**Visit and footwear**	**Mean**	**Difference**			p-values*		
			**Point est.**	**95% CI**		**Footwear-specific change**	**Change differs by footwear**	**Any change**
Foot Pain VAS	Visit 1 (Own)	37.8						
	Visit 2 (Asteroid)	20.0	−17.8	−44.0	8.3	0.19	0.47	0.028
	Visit 2 (Apollo)	34.6	−3.2	−29.4	22.9	0.81		
	Visit 2 (Helix)	34.7	−3.1	−33.3	27.0	0.84		
	Visit 2 (Cardio Zip)	16.0	−21.9	−34.5	−9.3	0.002		
Pain VAS	Visit 1 (Own)	35.7						
	Visit 2 (Asteroid)	45.1	9.4	−11.5	30.2	0.39	0.07	0.012
	Visit 2 (Apollo)	34.0	−1.7	−22.6	19.1	0.87		
	Visit 2 (Viper)	33.3	−2.4	−26.5	21.6	0.84		
	Visit 2 (Cardio Zip)	16.3	−19.4	−29.3	−9.6	0.001		
Patient global assessment VAS	Visit 1 (Own)	37.9						
	Visit 2 (Asteroid)	30.9	−7.1	−28.2	14.1	0.52	0.61	0.39
	Visit 2 (Apollo)	37.1	−0.8	−22.0	20.4	0.94		
	Visit 2 (Viper)	16.2	−21.7	−46.1	2.6	0.09		
	Visit 2 (Cardio Zip)	32.5	−5.4	−15.6	4.8	0.31		
HAQ-II	Visit 1 (Own)	0.8						
	Visit 2 (Asteroid)	0.7	−0.1	−0.5	0.2	0.38	0.07	0.016
	Visit 2 (Apollo)	1.0	0.2	−0.1	0.5	0.15		
	Visit 2 (Helix)	0.7	−0.1	−0.5	0.2	0.55		
	Visit 2 (Cardio Zip)	0.5	−0.3	−0.4	−0.1	0.002		
LFIS	Visit 1 (Own)	10.6						
	Visit 2 (Asteroid)	11.3	0.7	−2.6	4.0	0.68	0.37	0.03
(Impairment/footwear)	Visit 2 (Apollo)	9.1	−1.6	−4.8	1.7	0.35		
	Visit 2 (Viper)	8.2	−2.4	−6.2	1.4	0.22		
	Visit 2 (Cardio Zip)	8.1	−2.5	−4.1	−1.0	0.004		
LFIS	Visit 1 (Own)	15.6						
	Visit 2 (Asteroid)	10.9	−4.7	−9.5	0.2	0.07	0.46	0.11
(Activities/Participation)	Visit 2 (Apollo)	15.6	0.0	−4.8	4.9	1.00		
	Visit 2 (Viper)	11.0	−4.6	−10.2	1.0	0.12		
	Visit 2 (Cardio Zip)	13.7	−1.8	−4.1	0.4	0.12		
LFIS (total)	Visit 1 (Own)	26.2						
	Visit 2 (Asteroid)	22.6	−3.6	−10.8	3.5	0.33	0.80	0.064
	Visit 2 (Apollo)	24.5	−1.7	−8.8	5.5	0.65		
	Visit 2 (Viper)	19.1	−7.1	−15.4	1.2	0.11		
	Visit 2 (Cardio Zip)	21.8	−4.4	−7.7	−1.0	0.02		
LLTQ	Visit 1 (own)	28.6						
	Visit 2 (Asteroid)	33.3	4.6	0.1	9.1	0.05	0.08	0.11
	Visit 2 (Apollo)	24.6	−4.1	−8.6	0.4	0.09		
(Daily)	Visit 2 (Viper)	28.7	0.1	−5.1	5.2	0.98		
	Visit 2 (Cardio Zip)	29.8	1.1	−1.0	3.2	0.30		
LLTQ	Visit 1 (Own)	18.0						
	Visit 2 (Asteroid)	24.0	6.0	−1.9	13.8	0.15	0.11	0.11
	Visit 2 (Apollo)	13.8	−4.2	−12.1	3.6	0.30		
(Recreational)	Visit 2 (Viper)	21.3	3.2	−5.8	12.3	0.49		
	Visit 2 (Cardio Zip)	14.1	−3.9	−7.6	−0.2	0.05		

Note: The p-values address the following null hypotheses. Footwear specific change: tests against a null hypothesis of no change between visits 1 and 2 for the target footwear only; Change differs by footwear: tests against a null hypothesis of similar change between visits 1 and 2 for any footwear; any change: tests against a null hypothesis of no change between visits 1 and 2.

**Figure 3:**
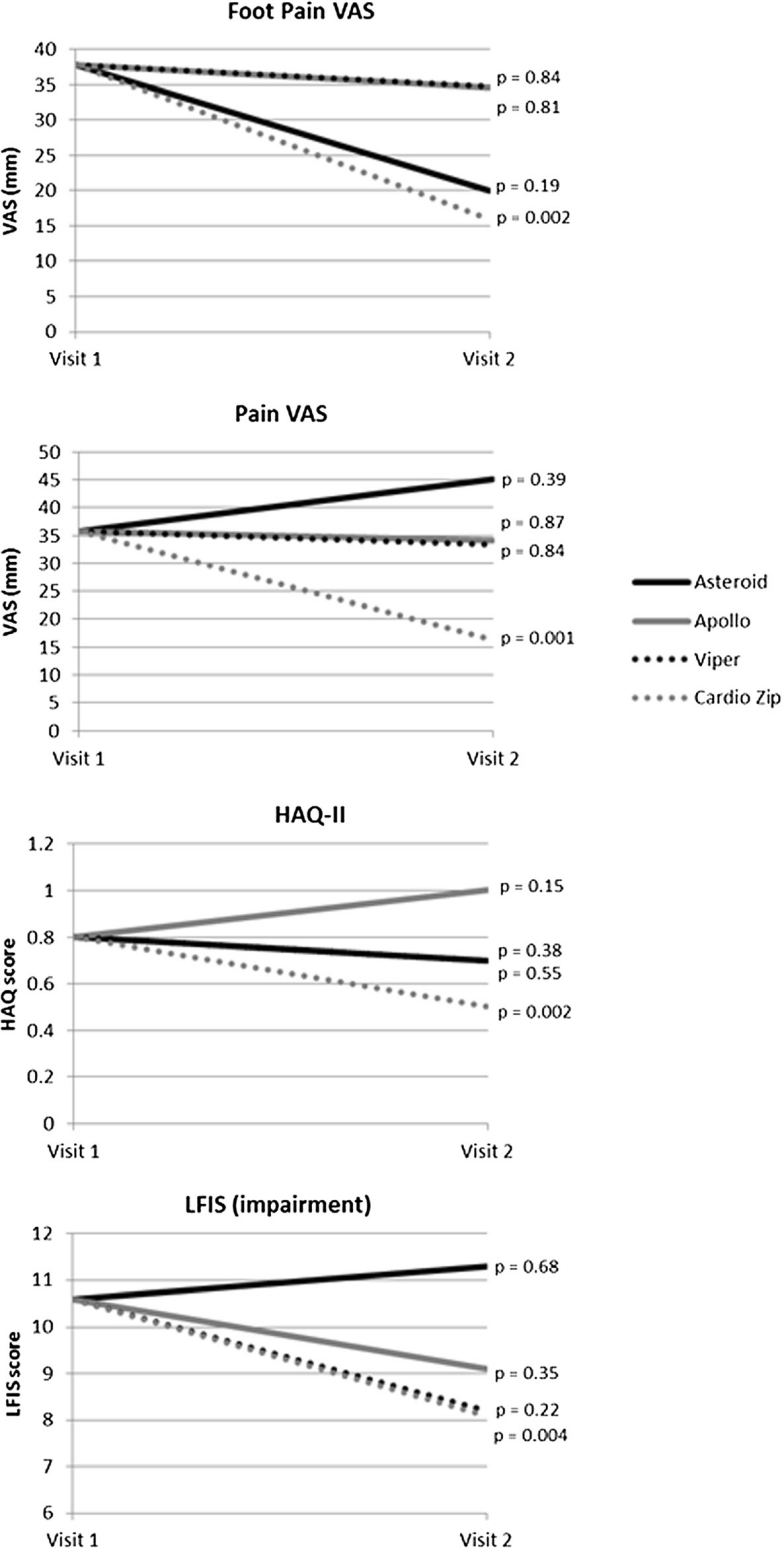
Patient reported outcomes from visit 1 to visit 2.

The patient-reported outcomes in the entire group at baseline and follow-up are shown in Table 3. After 8 weeks, there were significant improvements from baseline in the entire group in foot pain (p = 0.03), general pain (p = 0.012), HAQ II scores (p = 0.016) and LFISIF (p = 0.03). No significant differences were observed in the patient global assessment scores (p = 0.39), LFISAP (p = 0.11) activities of daily living using the Lower Limb Task Questionnaire (p = 0.11) or recreational activities using the Lower Limb Task Questionnaire (p = 0.11). We found significant improvements between the baseline visit scores (participants own shoes) and eight week scores for the Cardio Zip for foot pain (p = 0.002), general pain (p = 0.001), HAQ-II (p = 0.002) and LFISIF (p = 0.004). Improvements in foot pain or other patient reported outcomes were not observed in the other shoes. Figure 3 illustrates the differences of the four shoes for foot pain, general pain, HAQ-II and LFISIF.

## Discussion

This is the first intervention study examining the role of footwear in people with gout. In this study, most participants selected the Cardio Zip. We found that the Cardio Zip shoe significantly reduced foot pain, general pain and foot impairment over 8 weeks.

A number of features in the Cardio Zip shoe may be responsible for the reduction in pain and disability. The medial size zip closure in the Cardio Zip shoe enhances the ease of putting on and taking off the shoe. The Cardio Zip shoe also uses a dual-density midsole system to control motion. Barton [15] reported that the motion control properties of footwear are considered an important shoe feature in the management of patients with RA and musculoskeletal injuries. Another feature of the Cardio Zip shoe that may have reduced foot pain was the use of gel cushioning in the heel and forefoot regions to improve shock attenuation. This shoe element was not present in the other three shoe types. Dufour [19] reported that shoes that have softer out-soles, and mid-soles, or insoles that use elements of gel, foamed polyurethane, or air chambers can smooth (low pass filter) the shock wave associated with foot-strike. Finally, the Cardio Zip shoe midsole/outsole has a 'rocker' type system to create a smoother heel to toe transition during the gait cycle while maintaining both stability and comfort. Previous studies have reported that the toe rocker-soled shoe is thought to reduce pain by decreasing forefoot loading and promoting a normal heel-toe motion during gait [20-22]. Cho [20] reported that rocker-soled shoes with comfortable insoles may be enough to reduce foot pain and increase foot function for people with RA.

We found our participants wore the chosen shoes an average of 26 hours/week over the 8-week trial, demonstrating good adherence. Similar findings have been reported in RA studies using foot orthoses [23-27]. Patient acceptability and adherence are integral components of treatment success with footwear or foot orthoses interventions [28]. Williams [10] also reported significant improvements in foot pain associated with RA when patients were involved in the choice of their shoes, as compared to having a particular shoe prescribed to them. Given the number of factors that influence shoe choice in people with gout [12], it would be important that patients are educated in the key elements/concepts that they should look for when buying shoes in the retail sector. In agreement with our previous findings [12], the majority of participants’ own shoes would be regarded as old, and hence likely to be limited in their ability to control foot motion, and cushion the foot sufficiently.

We acknowledge that the study had limitations. The researcher collecting the outcome measures was not blinded to the shoes that the patient had chosen. However, the study protocol stated that the researcher should make no comments concerning the patient’s choice. The period over which shoes were worn was short and the shoes could be considered as being in a relatively new state after 8 weeks wear. A longer study would allow greater understanding of the effects of wear on the structural and mechanical properties of materials responsible for motion control and shock absorption, and the influence of these properties on long-term pain reduction. The high frequency of tophaceous disease reflects the hospital setting and the complexity of gout seen in Auckland, New Zealand particularly in the Māori and Pacific people. This may limit the generalisability of the findings to other populations with gout. Furthermore, it is unclear whether people with different stages of the disease would benefit from a similar intervention with footwear. Using the self-reported diaries to assess adherence was patient-reported only and therefore may be susceptible to bias/error.

## Conclusions

In conclusion, this is the first reported trial of a footwear intervention for people with gout. The findings suggest that a shoe with adequate motion control, cushioning, stability may reduce foot pain and musculoskeletal disability. Clinicians should consider footwear characteristics when advising people with gout on foot care. In addition to excellent control of serum urate concentrations using pharmacological treatment, footwear interventions may be of benefit in reducing foot pain and disability in people with gout. Future research is needed to observe the long-term impact of footwear on foot pain and musculoskeletal disability in people with gout. Future work should also consider cost-effectiveness analyses of footwear including studies to assess the impact of gout on indirect costs and quality of life on people with gout and their families.

## Competing interests

The authors declare that they have no competing interests.

## Authors’ contributions

SS carried out the data collection. AV performed the statistical analysis and KR, PMcN, PG, AV and ND contributed to the interpretation of the data. KR, PMcN, PG, AV and ND participated in the design of the study and drafting of the manuscript. All authors read and approved the final manuscript.

## Pre-publication history

The pre-publication history for this paper can be accessed here:

http://www.biomedcentral.com/1471-2474/14/278/prepub
